# Willing to pay to save the planet? Evaluating support for increased spending on sustainable development and environmentally friendly policies in five countries

**DOI:** 10.1371/journal.pone.0207862

**Published:** 2018-11-29

**Authors:** Todor Arpad

**Affiliations:** Political Science Department, National University for Political Studies and Public Administration, Bucharest, Romania; Universidad de Alicante, ITALY

## Abstract

While the 2016 Paris Agreement is in many ways an important attainment with the potential to represent a milestone in humanity’s path towards sustainable development, and avoid thus a potential calamitous and destructive future, the achievement of the goals set in the agreement is a long way off. This paper investigates one of the most important worldwide hurdles frustrating the implementation of the policies required to limit environmental degradation and limit pollution, namely the still insufficient public support for the necessary environmental policies and their associated cost. Using a comparative database generated through an experimental study on tax compliance and policy preferences run in five countries (USA, UK, Italy, Sweden and Romania), I will evaluate five explanatory models of the degree to which people support environmentally friendly policies by accepting higher tax burdens and increased collective solidarity.

## Introduction

While there is almost widespread scientific consensus that human activity generates many of the phenomena that lead to climate change, and that without proper action, climate change will irreversibly affect the entire planet, general public opinion is far from accepting these scientific facts and the need for urgent action. Technologically, rapidly eliminating the most important human-activity-based sources of climate change (greenhouse gases, pollution, deforestation) is possible. However, the significant financial efforts, the effect on GDP growth, [1] the consequential effect on the lifestyle of many people in developed countries, and the presence of a powerful minority of climate change deniers, all represent important political barriers to rapid and substantial policy changes.

This article contributes to the rich literature that investigates one of the most important hurdles to the rapid implementation of the required measures to prevent future catastrophic climate change: the limited public support for more environmentally friendly policies and the implications in terms of increased taxation. Using a database based on experimental research on tax compliance and policy preferences of mostly young people in five countries (USA, UK, Italy, Sweden, and Romania), I approach two research questions: (1) What are the factors that structure people’s willingness to support a state’s increased spending on environmentally friendly policies? and (2) To what extent is a preference for environmental policies associated with a predisposition toward more or less tax compliance under various institutional rules? The answer to the second research question can help us evaluate to what extent people’s willingness to support a state’s increased spending on environmentally friendly policies is backed by their willingness to pay their tax obligations.

An evaluation of the literature reveals five different research streams advancing alternative explanatory models relevant to the first research question: trust in state capacity; the Environmental Kuznets Curve; ideological preferences; ideological polarization; and the psycho-sociological characteristics model. Each of them is briefly reviewed in the following paragraphs. Following each short literature review, I extrapolate the hypothesis I will test using the experimental data used in this article. Subsequently, I briefly review the main explanatory models of tax compliance that will be used to answer the second research question.

Irrespective of the mix of factors that explain individual choices and preferences, all policies, especially environmental ones, depend on the capacity of institutions at various levels (local, national and international) to properly design them, enforce compliance and monitor their effects. A proper institutional design is vital not only in terms of implementing policies, but also in terms of generating public support for them. This is even more important in the case of the case of highly contentious policies, like the environmentally friendly policies. An investigation by Bernauer [2] identified four areas as key to understanding why solving global climate change issues is proving to be much more difficult than initially anticipated: (a) institutional designs that prevent the best outcomes in the international effort to mitigate climate change; (b) variations in climate policies at national and subnational levels; (c) driving forces of climate policy beyond the state, in particular civil society, the science–policy interface and public opinion; and (d) socio-political consequences of failing to avoid major climatic changes. Thus, despite the urgency of climate change action, the institutional structure through which cooperation takes place is inadequate and highly dependent on public support.

Within the same institutionalist approach, Bechtel and Scheve [3] reveal that support for climate agreements increases if the costs are low and are distributed across countries, respecting fairness principles, and if they are wide in terms of the number of countries that participate and are based on small sanctions for failure to comply. Pedersen [4] discusses the conditions under which eco-taxation transfers are legitimized by policy-makers as a useful tax instrument and shows the strong influence of administrative configuration bias and organized interest in shaping the chance of success. Also, Davidovic [5] discovered that while trust, both social and institutional, has a moderately positive effect on support for environmentally friendly policies in all countries, green values will have an enhanced effect in those countries where the quality of government is high. In other words, where institutions are effective in designing and implementing public policies, people are more likely to trust politicians when they are proposing new environmental taxes. Observing the Swedish case, Harring [6] also confirmed the finding that both political and interpersonal trust have a positive effect in fostering a favorable attitude towards the introduction of taxes on carbon dioxide emissions. Furthermore, using an experimental approach to investigate Britons’ willingness to pay environmental taxes for pollution, Fairbrother [7] found that the acceptance of new environmental taxes increases if an offset is offered, but only where there is a high level of institutional trust. Strengthening this argument, an evaluation into the effect of trust on people’s openness to accept environmental taxes by Kollmann and Reichi [8] shows that across 32 countries political trust increases the probability of the public accepting new environmental taxes from 17% to 23%. All these authors suggest that H1: people’s trust in the state’s capacity to negotiate and adequately implement policies is very important in determining people’s willingness to support spending on environmental policies.

Another line of inquiry, focusing on the link between development and environmentally friendly policies, is the inverted U relationship between evolution of income and environmental quality, a hypothesis based on Kuznets’ 1955 [9] income inequality–economic development model. The logic is that while in the initial stages of industrialization pollution rises abruptly, given the prioritization of investment in job-creating industrial output, subsequently, as a country develops, environmental concerns become more and more important. Even if the relevance of data used to support the model has been seriously questioned, and has been proven statistically significant across time and space only in the case of some types of air pollutant, the Environmental Kuznets Curve (EKC) model has been influential in the 1990s. Soumyananda reviewed the theoretical and empirical arguments in favor of the relevance of the EKC model, and grouped them into two main types of approach: arguments emphasizing the explanatory role of the transition toward a clean service economy and the development of stronger state institutions; and models emphasizing people’s higher preference for environmental quality once their income increases. [10] While there is no agreement in the literature about the income level turning point, one of the most important policy implications of the EKC model is that focusing on economic growth should be enough to generate increased protection for the environment at a later stage. [10] [11] Also, Castiglione et al. [12] found, using panel data, that efficient environmental taxation depends on a virtuous circle that connects efficient institutional enforcement and economic development. Within a similar approach, Muller and JhA [13] use data from US metropolitan areas to show that proper environmental policy implementation can limit the pollution increase from urbanization without having a negative effect on the economic productivity brought by increased urbanization. A comparative study by Atici on the relevance of the extended EKC model to explain the evolution of CO_2_ emissions per capita between 1980 and 2002 in Bulgaria, Hungary, Romania, and Turkey revealed that while development is associated with decreased emissions, increased openness to trade is not. [14] The main hypothesis that derives from this stream of research is that H2: the more developed a country is, the higher the support for environmental spending.

Particularly over the last decade, changes at the national level have influenced choices made by firms, with a growing focus on Corporate Environmental Responsibility or Environmental Corporate Social Responsibility (ECSR). The concept is defined by Mazurkiewics as “the duty to cover the environmental implications of the company’s operations, products and facilities; eliminate waste and emissions; maximize the efficiency and productivity of its resources; and minimize practices that might adversely affect the enjoyment of the country’s resources by future generations.”[15] Medarevic argues that an ECSR approach generates significant advantages for firms, especially when measures are taken voluntarily. [16] Rahman, aiming to systematize the literature in the field, identified the three most important dimensions of ECSR as governance, credibility, and environmental performance. [17] Another study identified e-customer welfare, community involvement and e-philanthropy as the most important dimensions of ECSR, and underlined the importance of customers’ perceptions of a firm’s environmental credentials, [18] a finding also backed by Lyon and Maxwell’s study. [19]

Beyond national and firm-level approaches, the individual level studies focus on the importance of individual values, interests and ideological preferences in shaping environmental preferences. While the presence of left-wing parties does not necessarily lead to lower pollution levels, since they promote policies to protect the industrial base, [20] people who prefer leftist values also support more environmentally friendly policies. In a review of the main findings of a two-decade cross-country analysis of environmental attitudes, Franzen and Vogl [21] showed that while the average concern with environmental issues increases as the wealth of a country increases, overall, environmental concerns have been slightly decreasing in the last two decades, especially in those countries that have experienced a lower level of economic growth. In countries such as the United States, Japan, and Germany, the number of people declaring they were willing to pay much higher prices or much higher taxes in order to protect the environment decreased on average by around 8% between 1993 and 2010. [21] The study also revealed that at the individual level, environmental concerns are determined by age, gender, education, income, ideological preferences, and post-material values. [21] Hypothesis H3 would be: the higher the support for left-wing policies, the higher the support for environmentally friendly policies.

One explanation for this change is that while in the 1970s support for the environment was seen as a trans-ideological issue in the United States, by the beginning of the 1990s, it had become a polarized one, a trend that has only grown since then both at the level of the US Congress and among the general public. [22] McCrigh et al.’s analysis of the relationship between people’s ideological view and their perception of climate change shows that while the US public is strongly divided by ideology, the divide is moderate in the old EU member states, and limited in the 11 post-communist countries. The limited partisan polarization in the post-communist countries is explained by the low saliency of these issues for the left–right divide.[23] [24] Hypothesis H4 is that the more the environmental issues are polarized in a country, the higher the explanatory power of ideology in that should be.

In a study of the ideological factors that determine climate change denial, Häkkinen and Akrami found out that while ideological orientation and preference for authoritarianism are important, social dominance orientation (SDO) has a stronger explanatory weight. Nevertheless, their experimental approach showed that climate change denial can be reduced across all type of ideological and value preference by properly communicating climate change evidence. [25] Also, the article by Harring et al. tested the interaction of ideology and personal values orientations’ effect on preference for environmentally friendly policies in the Swedish case. Their analysis showed that while these factors can act simultaneously, their effect is independent. [6] The analysis by Jagers et al. showed that left leaning persons are more environmentally friendly given their different perception of fairness and the effectiveness of various policy tools employed by state to achieve governmental goals. [26] The main hypothesis that can be tested within the data used in this article is that H5: people prone to more altruistic behavior should have a higher predisposition toward supporting more environmentally friendly policies.

In the second part of the literature review, I briefly present the most important explanatory models of tax compliance: trust in state capacity, cultural models and tax morale. The trust in institutional efficiency argument has been formulated in various forms in the last three decades, from Levi’s quasi-voluntary compliance argument [27] and Steinmo’s analysis of differences in tax systems [28] to the more recent studies that look at the attitudes towards the welfare state [29]. The perception that institutions efficiently use collected revenue increases both compliance and the willingness to accept new or higher taxes. The hypothesis deriving from this literature is that H6: the higher the trust in state capacity, the higher the level of tax compliance.

The cultural approach to tax compliance puts a fundamental emphasis on norms and values, especially the way social morality is defined in different countries. While in North European countries social, impersonal, autonomy-based norms are arguably dominant, southern or Latin American countries are argued to have more family-based norms [30–32]. This literature implies that H7: we should observe some important country-level effects on tax compliance, and that the more people support collectivistic norms, the lower the level of tax compliance. Pampel et al. [33] have nevertheless found that while individual preferences for fiscal responsibility, ideological values and belief in government competence affect tax compliance in an experimental setting, the *national culture* factors operationalized as country-level effects do not explain variations in tax compliance. Using data from Italy and the UK within the same research, Zhang et al. [34] discovered that when participants from the two countries were tested under experimental conditions using the same sets of institutions, their behavior went contrary to the cultural theories’ expectation, with Italians being much more tax compliant that their UK counterparts. Also, using the same database, Bruner et al. [35] found that gender is an important factor in shaping propensity for tax compliance, with women being more compliant both in intensive and extensive margins, despite the fact that men have a higher compliance when paying for public goods. Another approach on the factors that cause variation of tax compliance is the tax morale approach is provided by Torgler, [32] author that emphasizes the importance of an intrinsic, non-monetary motivation for tax compliance. The hypothesis deriving from the tax morale literature is that H8: the higher the belief in the importance of paying taxes, the higher tax compliance should be.

As in the case for preference for environmentally friendly policies, Lozza et al. [36] argue that support for left-wing policies, especially in terms of redistributive policies, is also associated with higher support for tax laws that would increase compliance. Based on the brief literature review I have extracted the most important hypotheses to be tested in explaining variations in the two dependent variables: support for more government spending, even if it might imply tax increases (H1–5) (models in Table 1), and tax compliance under various institutional structures (H6–8) (models in Table 2). The rest of the article is structured as follows. First, I present the methods and experimental protocol used to generate the data used for the analysis. Second, I discuss the characteristics of the data and the methodology used to analyze it. Subsequently I elaborate the results of the analysis and the discussion of the results, emphasizing the models that are proven to have a relevant explanatory power. In the Conclusions, I discuss the most important implications of the findings, and suggest some avenues for future research.

**Table 1:** Explanatory models for the preferences for more or less government spending on the environment.

		The countries on which the regression was run^a^					
		All	Italy	UK	US	Sweden	Romania
Explanatory models	Independent variables	b/se	b/se	b/se	b/se	b/se	b/se
H2 country level effects	Romania	-1.026***^b^					
		(0.20)					
	US	0.117					
		(0.16)					
	UK	-0.375*					
		(0.16)					
	Italy	-0.423*					
		(0.20)					
	Tax compliance	0.110	-0.138	0.220	0.072	-0.127	0.570*
		(0.09)	(0.24)	(0.17)	(0.17)	(0.22)	(0.27)
H1	Factor score–*believe in state competence*	0.108	0.298	0.017	-0.106	0.706*	0.048
		(0.11)	(0.31)	(0.24)	(0.19)	(0.29)	(0.26)
H3 H4	Factor score–*pro-redistribution ideology*	2.500***	1.835***	1.758***	3.052***	2.517***	1.620***
		(0.12)	(0.44)	(0.27)	(0.21)	(0.27)	(0.30)
	Factor score–*fiscal responsibility*	-0.073	-0.068	0.167	-0.412*	0.121	0.286
		(0.11)	(0.35)	(0.27)	(0.18)	(0.23)	(0.32)
H5 Psycho-social	SVO angle	0.004	0.012	-0.003	0.006	0.006	0.001
		(0.00)	(0.01)	(0.01)	(0.01)	(0.01)	(0.01)
	Risk acceptance	0.028	0.022	0.007	0.014	-0.008	0.084
		(0.02)	(0.07)	(0.05)	(0.04)	(0.06)	(0.05)
Controls	Age	-0.020**^e^	0.057*	-0.021*	-0.017	-0.025*	-0.010
		(0.01)	(0.03)	(0.01)	(0.02)	(0.01)	(0.02)
	Male	0.318**	0.669*	0.510*	0.386*	0.142	-0.042
		(0.10)	(0.28)	(0.22)	(0.18)	(0.24)	(0.24)
	Participated in other experiments?	0.195	-0.008	0.342	0.087	0.394	0.412
		(0.11)	(0.37)	(0.26)	(0.17)	(0.28)	(0.27)
	Studies economics?	0.155	0.749*	0.002	0.267	-0.449	-0.523
		(0.15)	(0.34)	(0.25)	(0.28)	(0.39)	(0.87)
	BIC	29550.6	3258.1	6617.2	8975.4	4837.4	4913.1
	N	13995.0	1656.0	2988.0	4527.0	2673.0	2151.0
	PseudoR-sq	0.2	0.2	0.1	0.2	0.3	0.2

The models were run using Ordered Logistic Regression with error terms clustered at the individual level using STATA 13.

^a^ The dependent variable had five possible choices (5 = Spend much more; 4 = Spend more; 3 = Spend the same as now; 2 = Spend less; 1 = Spend much less). The question contained the following note: “Remember that if you check ‘much more,’ it might require a tax increase to pay for it.”

^b^ Yellow shading underlines significant effects that are interpreted in the article.

The * symbol indicates a p<0.05 significance level.

The ** symbol indicates a p<0.01significance level.

The *** symbol indicates a p<0.001significance level.

**Table 2:** Explanatory models for the tax compliance for all scenarios and each separate scenario.

Explanatory models	Independent variables	All ^a^	y1	y2	y3	y4	y5	y6	y7	y8	y9
		b/se	b/se	b/se	b/se	b/se	b/se	b/se	b/se	b/se	b/se
Preference for more environmental spending		0.017*^b^	-0.001	0.023*	0.016*	0.012	0.011	0.010	-0.002	0.008	0.023*
		(0.01)	(0.01)	(0.01)	(0.01)	(0.01)	(0.01)	(0.01)	(0.01)	(0.01)	(0.01)
H2/H6 country level effects	Romania	0.083**	0.179***	0.117**	-0.010	-0.005	0.070	0.155***	0.110**	0.078*	0.067
		(0.03)	(0.04)	(0.04)	(0.04)	(0.04)	(0.04)	(0.04)	(0.04)	(0.04)	(0.04)
	US	0.018	0.094**	0.050	-0.031	-0.045	0.031	0.050	0.027	-0.010	0.053
		(0.02)	(0.03)	(0.03)	(0.03)	(0.03)	(0.03)	(0.03)	(0.03)	(0.03)	(0.03)
	UK	-0.031	-0.038	-0.073*	-0.092**	-0.077*	-0.026	0.009	0.001	-0.045	0.076*
		(0.02)	(0.03)	(0.03)	(0.03)	(0.03)	(0.03)	(0.03)	(0.03)	(0.03)	(0.03)
	Italy	0.050	0.120**	0.105**	0.063	0.123**	0.047	0.050	0.040	0.041	0.128***
		(0.03)	(0.04)	(0.04)	(0.04)	(0.04)	(0.04)	(0.04)	(0.04)	(0.04)	(0.04)
H6	Factor score–*believe in state competence*	0.031*	0.026*	0.026*	0.030**	0.019	0.040***	0.037***	0.031**	0.023*	0.040***
		(0.01)	(0.01)	(0.01)	(0.01)	(0.01)	(0.01)	(0.01)	(0.01)	(0.01)	(0.01)
H7	Factor score–*pro-redistribution ideology*	-0.002	-0.008	-0.011	-0.009	-0.003	-0.001	-0.005	-0.007	-0.007	-0.009
		(0.00)	(0.01)	(0.01)	(0.01)	(0.01)	(0.01)	(0.01)	(0.01)	(0.01)	(0.01)
H8	Factor score–*fiscal responsibility*	0.078***	-0.041**	-0.035**	-0.012	-0.017	0.001	-0.000	0.002	-0.014	-0.008
		(0.01)	(0.01)	(0.01)	(0.01)	(0.01)	(0.01)	(0.01)	(0.01)	(0.01)	(0.01)
	SVO angle	0.007***	0.007***	0.007***	0.007***	0.006***	0.008***	0.008***	0.009***	0.009***	0.006***
		(0.00)	(0.00)	(0.00)	(0.00)	(0.00)	(0.00)	(0.00)	(0.00)	(0.00)	(0.00)
	Risk acceptance	-0.021***	-0.030***	-0.027***	-0.016***	-0.021***	-0.024***	-0.011*	-0.021***	-0.025***	-0.017***
		(0.00)	(0.00)	(0.00)	(0.00)	(0.00)	(0.00)	(0.00)	(0.00)	(0.00)	(0.00)
	Age	0.003**	0.005***	0.003	0.001	0.001	0.004**	0.006***	0.004**	0.004**	0.000
		(0.00)	(0.00)	(0.00)	(0.00)	(0.00)	(0.00)	(0.00)	(0.00)	(0.00)	(0.00)
	Male	-0.128***	-0.168***	-0.158***	-0.099***	-0.091***	-0.121***	-0.159***	-0.154***	-0.118***	-0.110***
		(0.01)	(0.02)	(0.02)	(0.02)	(0.02)	(0.02)	(0.02)	(0.02)	(0.02)	(0.02)
	Participated in other experiments?	-0.079***	-0.118***	-0.087***	-0.057***	-0.059*	-0.077***	-0.041	-0.097***	-0.088***	-0.046*
		(0.01)	(0.02)	(0.02)	(0.02)	(0.02)	(0.02)	(0.02)	(0.02)	(0.02)	(0.02)
	Studies economics?	-0.056**	-0.046	-0.089**	-0.065*	-0.079*	-0.047	-0.011	-0.069*	-0.054	-0.050
		(0.02)	(0.03)	(0.03)	(0.03)	(0.03)	(0.03)	(0.03)	(0.03)	(0.03)	(0.03)
	constant	0.601***	0.582***	0.641***	0.811***	0.748***	0.578***	0.395***	0.603***	0.605***	0.730***
		(0.06)	(0.08)	(0.08)	(0.08)	(0.08)	(0.08)	(0.08)	(0.08)	(0.08)	(0.07)
	R-sqr	0.180	0.236	0.247	0.162	0.130	0.191	0.174	0.242	0.233	0.142
	dfres	1630	1412	1412	1412	1413	1413	1413	1415	1415	1415
	BIC	13458.8	1455.9	1347.3	1253.7	1448.7	1366.8	1489.5	1327.0	1199.7	1162.6
	N	14664.0	1427.0	1427.0	1427.0	1428.0	1428.0	1428.0	1430.0	1430.0	1430.0

The models were run using OLS regression with error terms clustered at the individual level using STATA 13.

^a^ The dependent variable had five possible choices (5 = Spend much more; 4 = Spend more; 3 = Spend the same as now; 2 = Spend less; 1 = Spend much less). The question contained the following note: “Remember that if you check ‘much more,’ it might require a tax increase to pay for it.”

^b^ Yellow shading underlines significant effects that are interpreted in the article.

The * symbol indicates a p<0.05 significance level.

The ** symbol indicates a p<0.01significance level.

The *** symbol indicates a p<0.001significance level.

## Methods and experimental protocol

The experimental protocol is described in detail in Zhang et al. [34], Brunner et al., [35] and D’Attoma et al., [37] and thus I will only present the main characteristics of the activity. The experimental sessions were organized from 2013 to 2017, with subjects recruited through public announcements and selected using ORSEE software [38] using similar procedures in all locations, in order to ensure similar demographic characteristics and unbiased selection mechanisms. In each country, the experiment was run in three to five different locations in different universities that had adequate physical infrastructure for the experiments: Bologna, Rome, and Milan in Italy; Oxford, London, Exeter, and Essex in the UK; Santa Cruz, California, Boulder, Colorado, Boone, North Carolina, Stony Brook, New York, and Honolulu, Hawaii in the United States; Stockholm and Gothenburg in Sweden; Bucharest, two different locations and Cluj in Romania. In practice, most participants were undergraduate students, and some (10%) recent graduates. Before the beginning of each session, participants received no information regarding the aim of the sessions or the comparative nature of the study. After their arrival, participants were randomly assigned an anonymous ID, were directed toward a computer station and were asked not to talk to the other participants until the end of the experimental session. Each experimental session took place for an average of 90 minutes and consisted of three stages of tax compliance, a Social Value Orientation stage, and a post-experimental survey.

The experimental protocol was translated in each language and doubly verified. All instructions were read by native speakers and the anonymity of decisions was explained and ensured by separators. The design aimed to avoid any national or group-level reputational bias and to simulate as genuinely as possible the real-life private decisions faced by taxpayers, and thus the standard vocabulary of tax administrations in each country was used.

The first three stages started with a five-minute clerical task consisting of copying information from a sheet onto a table. Based on their proficiency in executing the task, each participant earned a number of Experimental Currency Units (ECUs) (see Annex 1 for the average earnings by country and other descriptive statistics). Afterwards, each participant had to report their income from the clerical task with the formula “report your income for tax purposes” under nine different institutional scenarios, three for each stage. Each participant was informed during the instruction that there was a 5% cent audit probability, an audit that would fine them double the amount of money they under-reported. Whether a participant was audited was revealed only at the end of all stages in order to avoid any effect of the audit from one round to the next. In addition, the participants were unaware of others’ decisions and revenue. Thus, the main manipulation of the experiment consisted in asking for reporting decisions under different fiscal rules that mirrored the characteristics of the modern tax system in different countries. The instructions for each scenario were explained into detail and two examples were read for each scenario. The participants had the opportunity to ask for further information, in case they had any doubts about their options. The instructions read to participants for the nine scenarios were the following:

**Stage 1:** *Scenario 1*. Participants were told that the tax rate was 30% (no redistribution of tax revenue was mentioned). *Scenario 2*. The tax rate was 30%, but tax revenue was collected in a general fund, which was subsequently divided equally among all participants. *Scenario 3*. The tax rate remained at 30%, but all tax revenues in the general fund were doubled and then redistributed equally to all participants.

**Stage 2:** *Scenario 4*. A 10% tax rate, with tax revenues doubled and then redistributed. *Scenario 5*. A 30% tax rate, with tax incomes doubled and then redistributed. *Scenario 6*. A 50% tax rate, with tax revenues doubled and then redistributed.

**Stage 3:** *Scenario 7*. A progressive system taxed the top 10% of earners (as defined by their self-reported income) at 50%, the bottom 10% of earners at 10%, and the middle 80% of earners at 30%, with tax revenues doubled and then redistributed. *Scenario 8*. A marginal tax system taxed all subjects at 10% on the first 50 units of reported income, at 30% on the next 50 units, and at 50% on all reported income above 100. Taxed units were doubled and then redistributed. *Scenario 9*. A flat tax rate of 30%, with revenues doubled and then donated to charity [33].

The parameters that were altered were: (1) changing the sum participants received in return for the taxes they paid based on how much was collected altogether; (2) the tax rate; (3) the level of progressivity; (4) the institutional beneficiary of the taxes each participant paid (government or charity); and (5) the progressivity of benefits received by participants.

### Ethics statement

Experiments conducted in the present research were approved by the Ethics Committee at the University of Colorado, Boulder. Participants signed written informed consent prior to taking part in the study.

## Data and methodology description

Across the five countries, the sample from the experiment included 2036 subjects. Each single reporting decision was treated as a single variable with nine values for each person. The pooling of data provided a total N of 18324 (2036 x 9) but a small proportion of missing data on compliance, attitudes or other control variables reduced the sample to just 13995 decisions for the models in Table 1 and 14664 decisions for the models in Table 2.

The dependent variable for the first research question, *people’s willingness to support a state’s increased spending on environmentally friendly policies*, is operationalized through the following question from the post-experiment questionnaire: “Please indicate whether you would like to see more or less government spending in each area. Remember that if you check ‘much more’, it might require a tax increase to pay for it.” The variable had five possible choices: 5 = Spend much more; 4 = Spend more; 3 = Spend the same as now; 2 = Spend less; 1 = Spend much less. The same choices were also present for the other policy preference variables: health, police and law enforcement, education, military, pension, unemployment insurance, arts and culture. These variables are included in the factor scores. As the distance between the five choices cannot be assumed to be equal, but only ordered from low to high, the six models used to explain variation in this dependent variable (Table 1), a general one and five country-level models, were estimated using an ordered logistic regression.

The dependent variable for the second research question, the predisposition towards more or less tax compliance under various institutional rules, is operationalized through the observed compliance rate (calculated as the percentage of the reported income from the obtained income in each round) for each of the nine institutional scenarios of the experiment. Given that the distance between each choice is similar, the ten models in Table 2, a general one and one for each of the nine institutional scenarios, were estimated using an ordinary least square regression (OLS) with error terms clustered at the individual level.

To estimate the independent variable for H1 and H6, people’s trust in the state’s capacity, and H3, preference for left-wing policies, I have used the post-experimental survey in order to estimate factor scores that would group the participants’ most important policy preferences and attitudes. The factor score loads the responses to 25 questions and measures much more reliable and robust people’s attitudes and opinions than just single questions. The factor score procedure and syntax were designed by Pampel et al. [33] and applied on the same data set, except for Romania. For the purpose of this article, I re-run the procedure for all five countries, eliminating the preference for environmental spending from the analysis. The attitudinal questions were analyzed using orthogonal rotated principle components factor analyses and only three facto scores that had survey item loadings above .40 were retained (see Annex 2 for a detailed description).

The first factor scored, named *belief in government competence*, loads answers on questions regarding government’s competence, and indicates that even if the government manifests some shortcomings (e.g. too complex, tax rates too high, corruption, inefficiency), these are not enough to contest its overall competence, and do not justify tax evasion.

The *pro-redistribution ideology* loaded 15 items that reflected a high support for state spending on social programs, redistribution and other forms of government intervention. Also, scoring highly on this factor indicates a higher propensity to rank oneself as closer to the left of the political spectrum, with a belief in income equality and supporting the state’s responsibility for citizens’ well-being.

In contrast, the *fiscal responsibility* factor score gives a higher importance to not cheating tax obligations under any circumstances, the presence of intrinsic motivations for compliance and the need to treat tax evasion as a serious crime. Overall, higher scores on this factor indicate an intrinsic motivation for compliance.

While H2 will be tested through measuring existing country level effects, the independent variable for H5, people’s altruistic behaviors will be measured using the Social Values Orientation (SVO) [39]. After the tax compliance rounds, participants were required to make 15 allocation decisions to choose the distribution of tokens for themselves and another, anonymous, partner. The, procedure is detailed in Bruner et al., and was designed to identify the invidualistic vs. prosocial and altruistic motives in making the decisions. The tool allows for ranking people’s decision-making in a social context along a unique scale (ranked from competitive, individualist, pro-social to altruist).[35]

## Results and discussion

By way of background, data from the European Commission shows that Sweden, the UK and Romania all had revenue from environmental taxes of around 2.2% of GDP in 2014, while in Italy it reached 3.6%, among the highest in the European Union. While Romania had an implicit tax rate on energy of 50 Euro per ton of oil equivalent, it reached 210 in Sweden, 270 in the UK and topped 400 in Italy. [40] While these data are not matched against the level of tax evasion regarding environmental taxation, it showed that Italy is, by far, the most heavily taxed country from the group.

**Fig 1:**
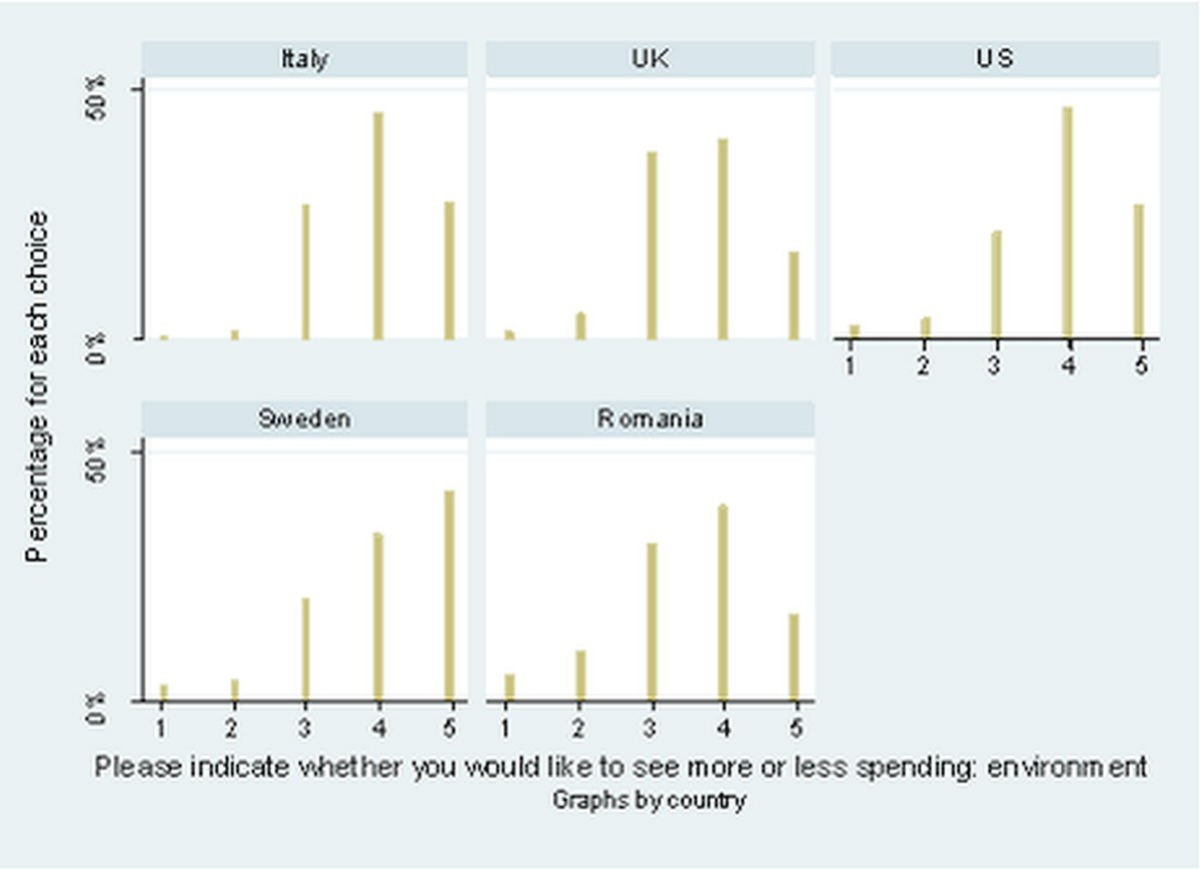
Preferences for more or less government spending on the environment. The five possible choices were: 5 = Spend much more; 4 = Spend more; 3 = Spend the same as now; 2 = Spend less; 1 = Spend much less. The question contained the following note: “Remember that if you check ‘much more’, it might require a tax increase to pay for it.”

Data distribution in Fig 1 shows a marked difference in country profiles. As discussed in the previous section, given the recruitment protocol, the average age of participants is significantly lower than that of the general adult population (see S1 Table), and thus the results are representative of the Millennial generation, and can be better used to understand the most probable future trends. While in Italy and the USA there are hardly any participants that prefer less and much less state spending for environmental policies, most participants prefer more spending. The percentage of participants who prefer spending levels to stay the same is relatively similar in all countries, except for Sweden. Romania has the highest number of people preferring less and much less spending and the lowest percentage who prefers much more spending, while Sweden is the opposite, being the only country where those that prefer much more spending represent the largest category.

**Fig 2:**
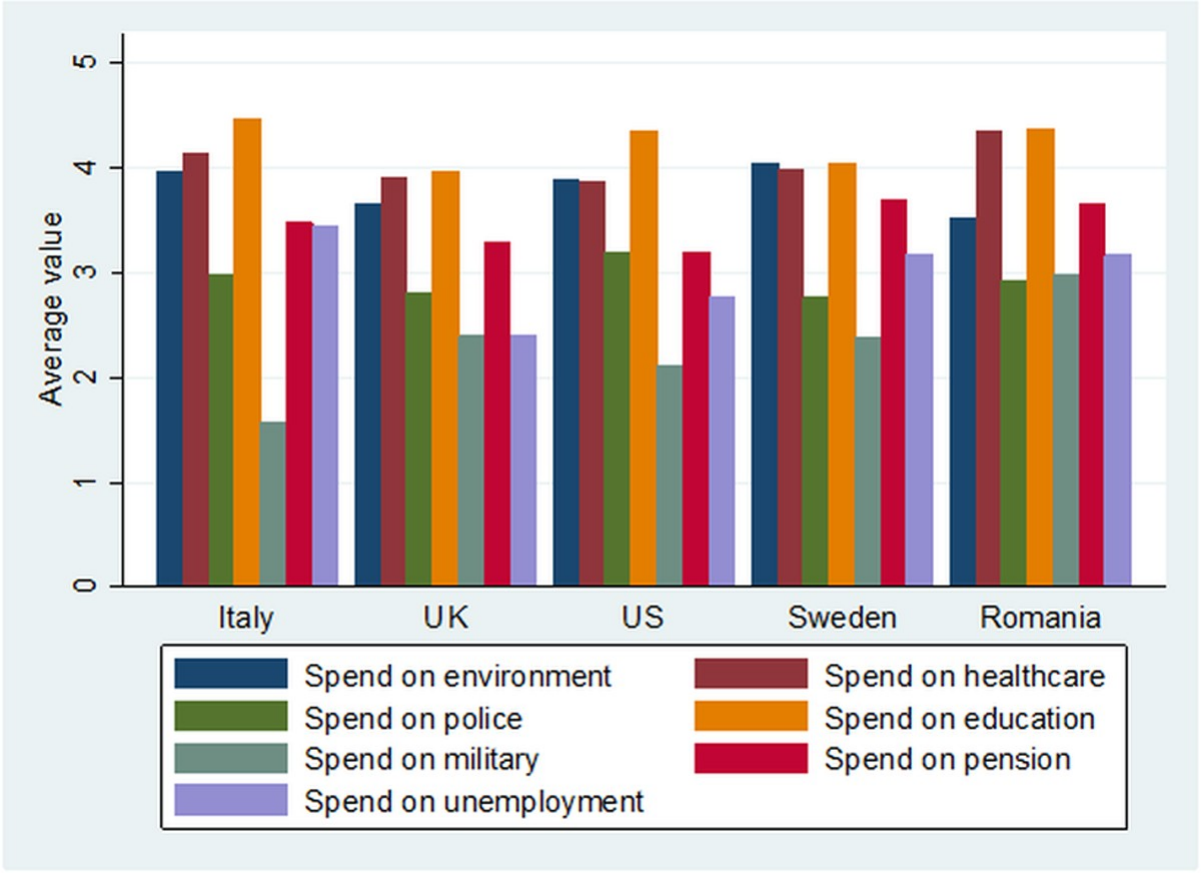
Country-level preferences for spending on various public policies. The five possible choices were: 5 = Spend much more; 4 = Spend more; 3 = Spend the same as now; 2 = Spend less; 1 = Spend much less. The question contained the following note: “Remember that if you check ‘much more’, it might require a tax increase to pay for it.”

When we compare the policy preferences for government spending in the area of environmental protection with the other policy preferences (Fig 2), the data reveals a slightly different profile for each country, although the overall rankings are relatively homogenous. Sweden is the only country where environmental spending tops any other policy area, this preference almost matched by preferences for education and health. The USA looks more similar to Sweden than expected, but with a preference for education spending outranking all other areas. In Italy, environmental policies are also among the top priorities. In all countries, preference for spending on unemployment, police and military spending comes last. All in all, despite the huge variations in terms of national development, position in the international arena and cultural traits, the ranking of young people’s policy preferences is much more homogenous than anticipated.

In Table 1 I present six regression models testing the explanatory capacity of the five models on the dependent variable, *people’s willingness to support a state’s increased spending on environmentally friendly policies*. The first model includes the data for all five countries, and the rest are separate models for each country. The regression coefficients confirm the descriptive data in Fig 1, showing the UK, Italy and especially Romania to have a significantly lower level of preference for environmental spending when compared to Sweden (the data for Sweden is used as a comparison for the other countries). Overall, the countries are arranged according to their level of development, with Romania, the poorest of the five countries, having the lowest level of environmental preferences, supporting H2 that is based on the EKC model. The level of tax compliance under various scenarios has no explanatory power, except for Romania. This finding indicates that the direction of causality from tax compliance towards preference for environmental spending is valid only in countries where the overall support for environmental spending is lower.

Given that the belief in state competency factor score is statistically insignificant in all countries except Sweden, where it has a positive effect on the dependent variable, H1, trust in state capacity, is not confirmed in general form, but confirms only Davidovic’s argument that institutional trust positively affects preference for environmentally friendly policies where the quality of government is high (as in Sweden).

Ideological preferences for pro-redistributive policies have the biggest positive impact on preferences for environmental spending in all six models, clearly supporting the argument (H3) that left-wingers are the most reliable environmentalists. Furthermore, hypothesis H4, regarding country-level political polarization is partially confirmed by interpreting the strength of the coefficient. While in Romania and Italy the explanatory power of left-wing preferences is lower, it is higher in the US, where the environmental policies are the most polarized. Nevertheless, the highest coefficient is in Sweden, which defies the expectations based on this hypothesis.

In contrast, the general model shows no effect of preference for fiscal responsibility on the preferences for environmentally friendly policies, with the country-level analysis showing that the factor score has a negative impact only in the USA. This result indicates that fiscal conservative people will reject more environmental spending only in countries where the environmental debate is politically polarized. The Social Value Orientation lacks explanatory power in all models, as well as the risk acceptances, clearly indicating that socio-psychological features are not relevant for explaining variation for environmentally friendly policies, and thus disproving H5. Among the control variables, the only one that is negatively significant is age, but only in the general models and in the UK and Sweden (where the effect is very small). Nevertheless, the fact that the age is statistically significant within a sample that has a limited age distribution, shows how important the change of preference from generation to generation is.

All in all, the statistically significant variables revealed by the first six models presented in Table 1 offer support for H2, as Sweden and the USA have the strongest preference for environmental spending, and Romania the weakest. H1 is valid only in cases where a high quality of government is present. H4, referring to the country-level political polarization and especially H3 referring to the individual level importance of ideology are both confirmed, with the individual level coefficients being several times higher than any other statistically significant coefficient.

In order to take advantage of the experimental data that tested people’s tax compliance under different institutional settings, in Table 2 I present a series of models aimed at answering the second research question. This answer is relevant in understanding to what extent a positive attitude toward environmentally friendly policies generates a propensity for tax compliance. This also allows us to understand the effect of different tax scenarios. Thus, the models presented in Table 2 are designed to test whether the preference for more or less governmental intervention and spending on environmental policies has any effect on how people react to various tax rule incentives, controlling for all alternative explanatory models of tax compliance. The first model explains variation in the compliance rate for all the nine scenarios, while y1 to y9 represent separate regression models for each of the nine scenarios. When disentangling this preference in each of the nine rounds we observe that the positive effects can be observed in only three distinct scenarios: in *Scenario 2*, where the tax rate is 30%, but tax revenues are collected in a general fund which is subsequently divided equally among all participants; in *Scenarios 3*, where the tax rate remained at 30%, but all tax revenues in the general fund were doubled and then redistributed equally to all participants; and in *Scenario 9* where there is a flat tax rate of 30%, with revenues doubled and then donated to charity. When compared with the other scenarios, where no statistically significant effect is observed, scenarios 2, 3 and 9 are peculiar in that they are flat taxation scenarios resulting in equal redistribution and state efficiency (the doubling of the amount) or redistribution though charity. These are effects coherent with the findings of the models in Table 1, showing that ideological effects are higher than any other explanatory variable. People who prefer environmentally friendly policies also react positively to tax policies aimed at redistribution. Nevertheless, the importance of redistribution and the preference for equal and not progressive taxation indicate an important change in the policy preferences of young people.

The models also show that when taking into account tax compliance for all nine rounds, the preference for more government spending has a statistically significant positive effect on tax compliance. When we control for preference for environmental spending, psycho-sociological features, SVO angle and risk acceptance, appear to be the most relevant in explaining variations in tax compliance, while ideological factors and country-level effects are absent, except for Romania. The belief in government competence is the only factor score that has a positive effect on compliance in the general model and in almost all scenario based models. This positive effect indicates that improving government’s perceived competence can improve tax compliance.

## Conclusions

In this article I have developed a novel angle of investigation into one of the most important hurdles still to be overcome in enacting the required measures to limit the catastrophic consequences of human activity on the environment. By analyzing how people from five countries react to various tax scenarios under experimental conditions and evaluating their policy preferences, I have tested the explanatory power several models extracted from the literature on the variation of two dependent variables: support for more government spending on the environment, even if it leads to tax increases, and tax compliance under various institutional structures.

This investigation brings several contributions to the literature. First of all, the public policy preferences show that, despite variations among the five countries, support for *more government spending*, *even if it might lead to tax increases*, is dominant among young people from these countries. The analysis showed the importance of values, and most importantly, left-wing ideological preferences, for the support for environmentally friendly policies. The analysis also revealed the positive effect of support for environmentally friendly policies on tax compliance. Given that opinion pool analysis showed that young people across developed countries tend to be more left leaning, the findings of this analysis indicate that support for environmentally friendly policies is likely to steadily increase in the future and become dominant.

The fact that belief in government competence does not have a significant explanatory power, except in the case of a high quality of government, could be interpreted as a negative finding since it underscores the limitations of a government’s capacity to foster support for these policies. Nevertheless, this finding indicates that as the quality of government increases, further support for environmentally friendly policies could be mobilized. The fact that the EKC and the political polarization models have an explanatory power, while controlling for so many powerfully individual level variables, is an important finding. One of the most important implications from this finding is that understanding the evolution of support for environmentally friendly policies requires a multiple level analysis. The policy implication of this analysis is that designing and advocating environmentally friendly policies should be customized to the specificity of each country regarding its level of development, political polarization regarding environmental issues and the association of these policies with people’s ideological preferences.

This article has innovatively connected the tax compliance literature with the literature on support for the environment. While the level of tax compliance measured under experimental conditions does not explain the variation in support for environmentally friendly policies, people’s support for environmentally friendly policies is shown to increase compliance in those scenarios that involve tax redistribution. The positive association between the preference for flat, not progressive taxation, indicates an important change in the policy preferences of young people and also underscores the importance of how the framing of these policies could be improved to make them more attractive, especially in order to improve compliance in the area of environmental taxation. Thus, future research should focus on how the framing of environmental policies and taxes could be improved to make them more acceptable, especially taking into consideration different ideological policy preferences.

## Supporting information

S1 Table Mean values and standard errors by country for the variables used in the regression models. (DOCX)

S2 Table Factor loadings and items for three factor scores used in the regression models. (DOCX)
