# Correction: Willing to pay to save the planet? Evaluating support for increased spending on sustainable development and environmentally friendly policies in five countries

**DOI:** 10.1371/journal.pone.0216571

**Published:** 2019-05-03

**Authors:** Arpad Todor

The author’s name appears incorrectly. The correct name is Arpad Todor. The correct citation is: Todor A (2018) Willing to pay to save the planet? Evaluating support for increased spending on sustainable development and environmentally friendly policies in five countries. PLoS ONE 13(11): e0207862. https://doi.org/10.1371/journal.pone.0207862.
