# Supplementary material for: Willing to pay to save the planet? Evaluating support for increased spending on sustainable development and environmentally friendly policies in five countries
Source: PLoS One. 2018 Nov 29;13(11):e0207862. doi: 10.1371/journal.pone.0207862 (PMC6264805; doi:10.1371/journal.pone.0207862)
Supplement: S1 Table — (DOCX) [file pone.0207862.s001.docx]

**S1 Table. Mean values and standard errors by country for the variables used in the regression models**

| **Country** | **Mean** | **Std. Err.** | **Country** | **Mean** | **Std. Err.** | **Country** | **Mean** | **Std. Err.** |
| --- | --- | --- | --- | --- | --- | --- | --- | --- |
| **Average ECU earnings in the tax section of the experiments** | | | **Factor score – *believe in state competence*** | | | **Preference for sepnding on the environment** | | |
| Italy | 1,063 | 5.40 | Italy | -0.16019 | 0.012416 | Italy | 3.94 | 0.02 |
| UK | 1,151 | 4.80 | UK | 0.052852 | 0.009216 | UK | 3.584 | 0.02 |
| US | 1,176 | 4.08 | US | -0.15314 | 0.007486 | US | 3.877 | 0.01 |
| Sweden | 1,065 | 4.63 | Sweden | 0.327718 | 0.0107 | Sweden | 4.04 | 0.02 |
| Romania | 913 | 4.54 | Romania | -0.09784 | 0.011164 | Romania | 3.526 | 0.02 |
|  |  |  |  |  |  |  |  |  |
| **Average compliance in all rounds – y** | | | **Risk** |  |  | **SVO angle** |  |  |
| Italy | 65% | 0.0100 | Italy | 5.201 | 0.057407 | Italy | 15.95 | 0.36 |
| UK | 52% | 0.0081 | UK | 6.298 | 0.038494 | UK | 16.62 | 0.26 |
| US | 68% | 0.0160 | US | 5.97 | 0.03254 | US | 25.71 | 0.21 |
| Sweden | 67% | 0.0183 | Sweden | 5.701 | 0.042203 | Sweden | 27.54 | 0.25 |
| Romania | 74% | 0.0079 | Romania | 6.232 | 0.053434 | Romania | 25.6 | 0.30 |
|  |  |  |  |  |  |  |  |  |
| **Factor score – *pro-redistribution ideology*** | | | **Age** |  |  | **Participated in other experiments?** | | |
| Italy | 0.1865586 | 0.01 | Italy | 24 | 0.089749 | Italy | 80% | 0.009647 |
| UK | -0.1978453 | 0.01 | UK | 24.5 | 0.206913 | UK | 83% | 0.006803 |
| US | -0.0844318 | 0.01 | US | 21.3 | 0.074834 | US | 56% | 0.007384 |
| Sweden | 0.1654753 | 0.01 | Sweden | 27.8 | 0.164689 | Sweden | 73% | 0.00859 |
| Romania | 0.0295893 | 0.01 | Romania | 216 | 0.097202 | Romania | 27% | 0.009653 |
|  |  |  |  |  |  |  |  |  |
| **Factor score – *fiscal responsibility*** | | | **male** |  |  | **Study economy** | | |
| Italy | 0.2237948 | 0.01 | Italy | 51% | 0.012284 | Italy | 27% | 0.011003 |
| UK | 0.0043694 | 0.01 | UK | 56% | 0.009082 | UK | 23% | 0.007757 |
| US | -0.0516425 | 0.01 | US | 42% | 0.007357 | US | 10% | 0.00461 |
| Sweden | 0.1433041 | 0.01 | Sweden | 55% | 0.009624 | Sweden | 13% | 0.006541 |
| Romania | -0.292209 | 0.01 | Romania | 46% | 0.010758 | Romania | 3% | 0.003885 |
