# Supplementary material for: Willing to pay to save the planet? Evaluating support for increased spending on sustainable development and environmentally friendly policies in five countries
Source: PLoS One. 2018 Nov 29;13(11):e0207862. doi: 10.1371/journal.pone.0207862 (PMC6264805; doi:10.1371/journal.pone.0207862)
Supplement: S2 Table — (DOCX) [file pone.0207862.s002.docx]

**S2 Table . Factor loadings and items for three factor scores used in the regression models**

| **Pro-redistribution ideology** | **Fiscal responsibility** |
| --- | --- |
| .75 More government unemployment spending | .70 Paying taxes is a fundamental duty of citizenship |
| .72 Government should take more responsibility | .62 Never justifiable to cheat on taxes |
| .69 Incomes should be more equal | .60 Paying taxes funds useful and important services |
| .66 Self-placement on the left of the political scale | .59 Should pay taxes even if they are unfair |
| .62 Taxes are too low for those with high income | .58 Disagree: It is right not to pay taxes if they are unfair |
| .60 More government health spending | .54 Should pay taxes to help the most vulnerable |
| .57 Competition is harmful | .53 Not paying taxes is one of the worst crimes |
| .57 More government culture spending | .47 Never justifiable to cheat on claiming benefits |
| .54 Increase government ownership |  |
| .53 More a person earns, the higher taxes should be | **Belief in government competence** |
| .48 More government pension spending | .66 Disagree: Pay if had some control over spending |
| .48 More government education spending | .62 Disagree: Cheat because politicians are corrupt |
| .45 Better life comes from luck and connections | .58 Disagree: Pay if government worked more efficiently |
| .42 It is right to pay taxes to help the most vulnerable | .56 High taxes don't force individuals to evade taxes |
|  | .56 Disagree: Cheat because the tax rates are too high |
|  | .49 Disagree: Spend tax money where taxes are collected |
|  | .48 Tax money not used to support lazy people |
|  | .47 Taxes are too low on middle income groups |
|  | .44 Disagree: People afraid of making a mistake in taxes |
|  | .41 Disagree: Cheat because tax system is too complex |
